# Using higher cut-off values to diagnose acute myocardial infarction in patients with elevated hs-cTnT

**DOI:** 10.7555/JBR.38.20240364

**Published:** 2025-05-21

**Authors:** Tian Wu, Jiaqi Chai, Chunyue Tan, Zhiwen Tao, Hui Yong, Zhenyu Lin, Xiaoxuan Gong, Kun Liu, Lei Xu, Qin Wang, Shenqi Jing, Jiani Xu, Hui Zhou, Tao Li, Liang Yuan, Bo Chen, Fang Wang, Ruxing Wang, Yun Liu, Chunjian Li

**Affiliations:** 1 Department of Cardiology, the First Affiliated Hospital of Nanjing Medical University, Nanjing, Jiangsu 210029, China; 2 Center for Data Management, the First Affiliated Hospital of Nanjing Medical University, Nanjing, Jiangsu 210029, China; 3 Department of Medical Informatics, School of Biomedical Engineering and Informatics, Nanjing Medical University, Nanjing, Jiangsu 211166, China; 4 Institute of Medical Informatics and Management, Nanjing Medical University, Nanjing, Jiangsu 211166, China; 5 Jiangsu Province Engineering Research Center of Chronic Disease Big Data Application and Smart Healthcare Service, Nanjing, Jiangsu 210029, China; 6 Shanghai Synyi Medical Technology Co., Ltd., Shanghai 200000, China; 7 Department of Cardiology, the Affiliated Wuxi People's Hospital of Nanjing Medical University, Wuxi People's Hospital, Wuxi Medical Center, Nanjing Medical University, Wuxi, Jiangsu 214023, China

**Keywords:** acute myocardial infarction, high sensitivity cardiac troponin T, electrocardiogram, ST-segment elevation myocardial infarction, renal dysfunction

## Abstract

It is often challenging to diagnose acute myocardial infarction (AMI) in patients with elevated high-sensitivity cardiac troponin T (hs-cTnT) before observing a significant rise and/or fall in hs-cTnT. The current study aimed to identify an optimal cut-off to rule in AMI. A total of 76411 patients with elevated hs-cTnT were included. The predictive cut-off values for diagnosing ST-segment elevation myocardial infarction (STEMI) and non-ST-segment elevation myocardial infarction (NSTEMI) were assessed using the area under the receiver operating characteristic curve (AUC). Among the patients, 50466 (66.0%) had non-cardiac diseases, 25945 (34.0%) had cardiac diseases, and 15502 (20.3%) had AMI, including 816 (1.1%) with STEMI and 14686 (19.2%) with NSTEMI. The median hs-cTnT level was 3788.0 ng/L in STEMI patients and 67.2 ng/L in NSTEMI patients. The optimal cut-off for diagnosing STEMI was 251.9 ng/L, with a sensitivity of 90.7%, specificity of 86.5%, and an AUC of 0.942; the optimal cut-off for diagnosing NSTEMI was 130.5 ng/L, with a sensitivity of 40.9%, specificity of 83.8%, and an AUC of 0.638. Collectively, optimizing the cut-off values for diagnosing STEMI and NSTEMI to 251.9 ng/L and 130.5 ng/L, respectively, demonstrated high accuracy in a large cohort of Chinese patients with elevated hs-cTnT.

## Introduction

Acute myocardial infarction (AMI), the most severe manifestation of coronary atherosclerotic disease, is a major cause of morbidity and mortality worldwide^[1–2]^. It occurs primarily because of partial or complete occlusion of the coronary arteries, leading to acute and sustained ischemia and hypoxia in portions of the myocardium, ultimately resulting in localized myocardial necrosis^[3]^. In clinical practice, the early and accurate recognition of AMI is crucial for accelerating effective reperfusion therapy, thereby reducing mortality and improving patient prognosis.

Among various biomarkers used in the diagnosis of AMI, high-sensitivity cardiac troponin T (hs-cTnT) has emerged as a cornerstone. Cardiac troponin T (cTnT), a component of the contractile apparatus of myocardial cells, is released into the blood after myocardial injury or infarction^[4]^. Hs-cTnT has become one of the primary indicators for diagnosing AMI and for risk stratification. Because of its higher accuracy compared with traditional biomarkers, hs-cTnT has been widely adopted in clinical practice^[5]^.

According to the fourth universal definition of AMI, a rise and/or fall in cTnT values, with at least one value above the 99th percentile upper reference limit (URL), is necessary to diagnose AMI^[3]^. However, it is challenging to diagnose AMI before a rise and/or fall in hs-cTnT values can be observed, particularly in patients with atypical symptoms and non-specific electrocardiogram (ECG) changes. Moreover, when referring to the current 99th percentile URL, elevated hs-cTnT levels have been observed in many cardiac and non-cardiac diseases other than AMI^[6–8]^. Therefore, thresholds above the 99th percentile have been proposed to improve the specificity and to accelerate the rule-in of myocardial infarction^[8–9]^.

In the current study, we aimed to identify an optimal cut-off value of hs-cTnT to rule in AMI in a large cohort of patients with elevated hs-cTnT.

## Subjects and methods

### Study design and participants

A single-center retrospective study was conducted among hospitalized patients at the First Affiliated Hospital of Nanjing Medical University, Nanjing, China. Patients with elevated peak hs-cTnT levels were included. The exclusion criteria were patients who underwent major cardiac surgeries during hospitalization.

### Data extraction and acquisition

Inpatients' data were obtained from the hospital's clinical data repository, which integrates patients' baseline characteristics, laboratory results, clinical diagnosis, and treatment information during the index hospitalization.

Patients hospitalized between January 1, 2015, and May 31, 2023, with hs-cTnT levels above 14.0 ng/L were included. If a patient had multiple hs-cTnT results during the index hospitalization, the maximum value was adopted. Data extraction was conducted to obtain the patients' primary discharge diagnoses, and the patients were categorized according to their diagnoses.

### Laboratory tests

Hs-cTnT levels were routinely measured upon patients' admission and again on the following morning for AMI patients. If a patient developed acute chest pain or worsening symptoms after admission, hs-cTnT levels were measured immediately, followed by a series of hs-cTnT tests performed at the discretion of the attending physicians.

Blood samples were collected in ethylene diamine tetraacetic acid (EDTA)-plasma tubes. Hs-cTnT levels were measured using Elecsys hs-cTnT STAT assay on the Cobas e601 module (Roche Diagnostics, Mannheim, Germany). The 99th percentile URL was set at 14.0 ng/L. According to the manufacturer's instructions, the measuring range of the assay was 3.0–10000.0 ng/L (defined as the limit of detection to the maximum value of the calibration curve).

### Definitions

AMI was defined as a rise and/or fall in cTnT values, with at least one value above the 99^th^ percentile URL, along with at least one of the following criteria: (1) Symptoms of myocardial ischemia; (2) New ischemic ECG changes—based on which AMI was further classified into ST-segment elevation myocardial infarction (STEMI) and non-ST-segment elevation myocardial infarction (NSTEMI); (3) Development of pathological Q waves; and (4) Imaging evidence of new loss of viable myocardium or new regional wall motion abnormality^[3,10–11]^.

Renal dysfunction was defined to include both acute and chronic types, and the diagnostic criteria followed international guidelines^[12–14]^. Chronic kidney disease was defined as abnormalities of kidney structure or function that persist for more than three months and involved at least one of the following: albuminuria, urine sediment abnormalities, electrolyte and other abnormalities caused by tubular dysfunction, abnormal renal histology, abnormal renal imaging, a history of kidney transplantation, and/or a decreased glomerular filtration rate of less than 60 mL/min per 1.73 m^2^ for more than three months^[12–13]^. The diagnosis of acute kidney injury was established when any of the following criteria were met: an increase in serum creatinine by ≥ 0.3 mg/dL within 48 h, an increase in serum creatinine to ≥ 1.5 times the baseline within the previous seven days, or urine volume ≤ 0.5 mL/(kg·h) for six hours^[14]^.

### Statistical analysis

Normally distributed continuous variables were presented as mean ± standard deviation. Non-normally distributed continuous variables were presented as the median and interquartile range (IQR). The optimal cut-off point of hs-cTnT with the highest discriminating power was determined by receiver operating characteristic (ROC) curve analysis. The predictive value of the cut-off was assessed by the area under the ROC curve (AUC). The AUC was calculated from the entire study population, including both AMI and non-AMI cases. A two-tailed *P *value < 0.05 was considered statistically significant. All analyses were performed using SPSS 24.0 (SPSS Inc., Chicago, IL, USA) and MedCalc 19.7 (MedCalc Software Ltd., Ostend, Belgium).

## Results

### Baseline characteristics

**Figure 1:**
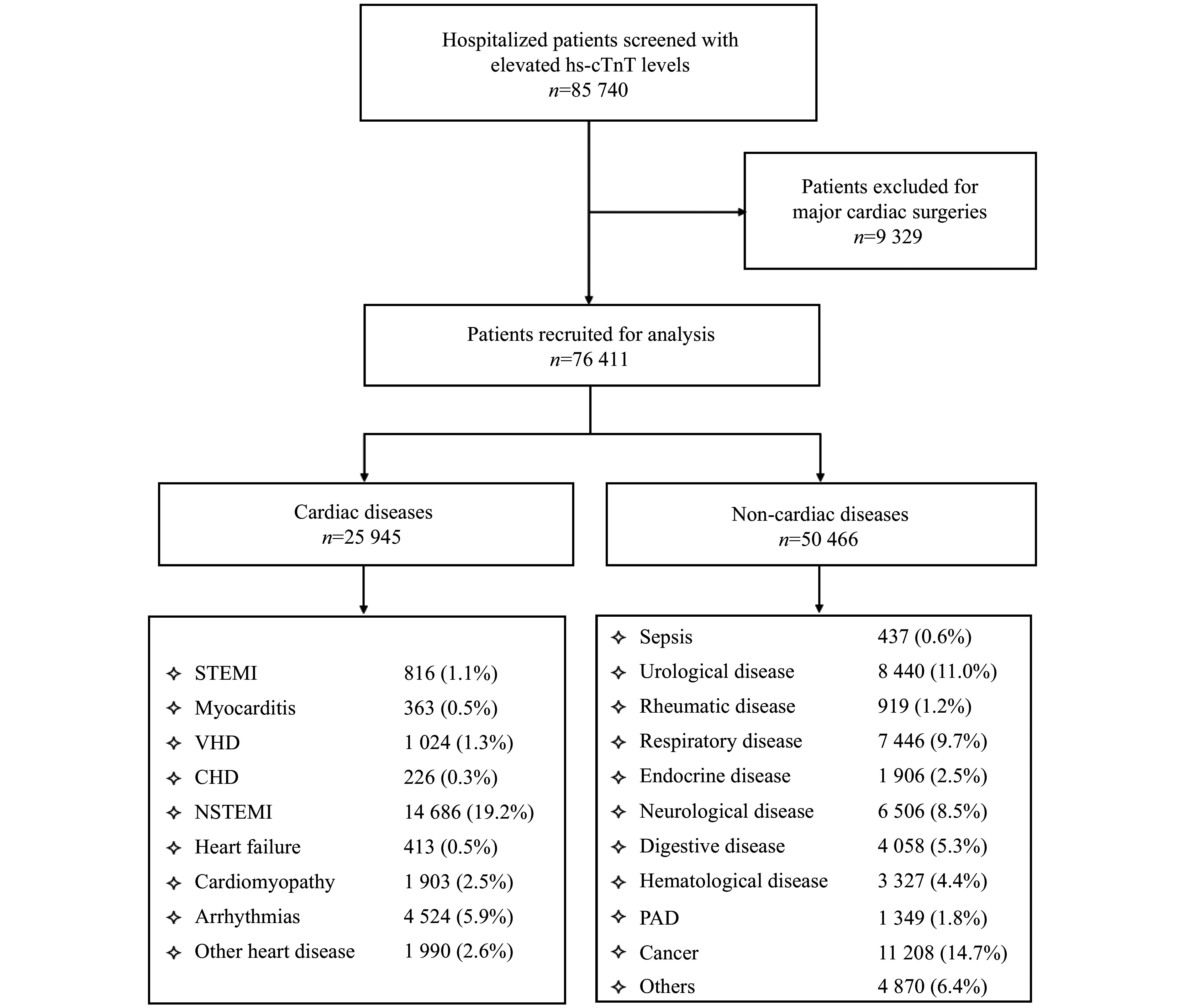
Study flowchart. Abbreviations: CHD, congenital heart disease; hs-cTnT, high-sensitivity cardiac troponin T; NSTEMI, non-ST-segment elevation myocardial infarction; PAD, peripheral arterial disease; STEMI, ST-segment elevation myocardial infarction; VHD, valvular heart disease.

**Figure 2:**
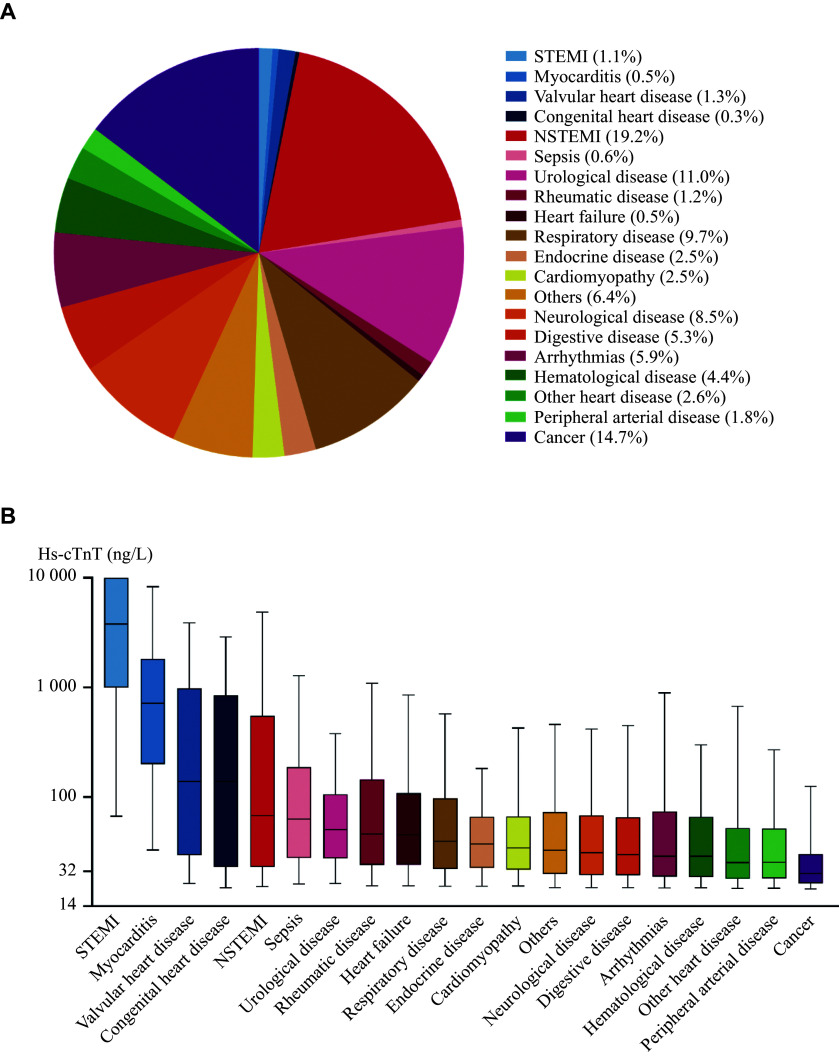
Distribution of diseases with different elevated hs-cTnT levels. A: Pie chart of the disease distribution in patients with elevated hs-cTnT levels. B: Distribution of diseases in patients with different hs-TnT levels presented as median and interquartile range. The whiskers extend to the 5th and 95th percentiles. Abbreviations: hs-cTnT, high-sensitivity cardiac troponin T; NSTEMI, non-ST-segment elevation myocardial infarction; STEMI, ST-segment elevation myocardial infarction.

A total of 85740 patients with elevated hs-cTnT were screened, among whom 9329 who underwent major cardiac surgery were excluded. As a result, 76411 patients were included in the analysis (***Fig. 1***). The patients' baseline characteristics are shown in ***Table 1*** and ***Fig. 2A***.

**Table 1:** Clinical characteristics

Characteristics	*n* (%)	Age (years)	Male (%)	Hs-cTnT (ng/L)
STEMI	816 (1.1)	63.5±13.8	81.5	3788.0 (993.5–10000.0)
Myocarditis	363 (0.5)	34.0±16.0	59.3	715.8 (199.5–1819.0)
VHD	1024 (1.3)	66.0±13.3	54.6	138.7 (29.4–980.7)
CHD	226 (0.3)	45.6±17.5	43.8	138.6 (22.9–846.0)
NSTEMI	14686 (19.2)	68.1±12.6	75.3	67.2 (22.9–548.9)
Sepsis	437 (0.6)	64.5±15.2	57.2	62.5 (27.7–186.9)
Urological disease	8440 (11.0)	60.5±17.5	65.7	50.4 (27.5–105.5)
Rheumatic disease	919 (1.2)	55.8±17.1	31.3	45.9 (23.9–144.4)
Heart failure	413 (0.5)	67.7±16.4	61.0	44.9 (24.0–108.0)
Respiratory disease	7446 (9.7)	75.3±14.9	69.0	39.4 (22.0–96.8)
Endocrine disease	1906 (2.5)	58.6±16.9	62.7	37.1 (22.5–65.7)
Cardiomyopathy	1903 (2.5)	58.8±14.8	76.5	34.0 (21.6–66.3)
Others	4870 (6.4)	66.4±17.0	62.8	32.5 (19.8–72.6)
Neurological disease	6506 (8.5)	70.8±15.7	67.9	30.9 (19.4–67.8)
Digestive disease	4058 (5.3)	70.5±14.4	65.5	29.7 (19.2–65.2)
Arrhythmias	4524 (5.9)	67.8±15.2	60.8	28.7 (18.7–73.3)
Hematological disease	3327 (4.4)	65.2±14.4	67.3	28.6 (18.5–65.7)
Other heart diseases	1990 (2.6)	67.3±18.2	66.1	25.1 (17.9–51.9)
PAD	1349 (1.8)	73.6±11.1	72.2	24.1 (17.8–45.8)
Cancer	11208 (14.7)	69.6±10.3	79.9	19.9 (16.2–29.9)
Total	76411	67.4±15.4	69.5	33.9 (19.6–97.7)
Values are presented as numbers and percentages (%), mean ± standard deviation, or median and interquartile range. Abbreviations: CHD, congenital heart disease; hs-cTnT, high-sensitivity cardiac troponin T; NSTEMI, non-ST-segment elevation myocardial infarction; PAD, peripheral arterial disease; STEMI, ST-segment elevation myocardial infarction; VHD, valvular heart disease.				

### Hs-cTnT concentrations and distribution

The median value of hs-cTnT was 33.9 ng/L (IQR: 19.6–97.7 ng/L). Thirty-four percent of the patients were diagnosed with cardiac diseases, while 66.0% had non-cardiac diseases. The cardiac disease cases included STEMI (1.1%), NSTEMI (19.2%), myocarditis (0.5%), valvular heart disease (VHD; 1.3%), congenital heart disease (CHD; 0.3%), heart failure (0.5%), cardiomyopathy (2.5%), arrhythmias (5.9%), and other heart diseases (2.6%). The non-cardiac disease cases included sepsis (0.6%), urological disease (11.0%), rheumatic disease (1.2%), respiratory disease (9.7%), endocrine disease (2.5%), neurological disease (8.5%), digestive disease (5.3%), hematological disease (4.4%), peripheral arterial disease (1.8%), cancer (14.7%), and others (6.4%).

The diagnostic distribution across hs-cTnT levels is shown in ***Table 1*** and ***Fig. 2B***. Among all diagnoses, STEMI had the highest median hs-cTnT value of 3788.0 ng/L (IQR: 993.5–10000.0 ng/L), followed by myocarditis (715.8 ng/L [IQR: 199.5–1819.0 ng/L]), VHD (138.7 ng/L [IQR: 29.4–980.7 ng/L]), CHD (138.6 ng/L [IQR: 22.9–846.0 ng/L]), NSTEMI (67.2 ng/L [IQR: 22.9–548.9 ng/L]), heart failure (44.9 ng/L [IQR: 24.0–108.0 ng/L]), cardiomyopathy (34.0 ng/L [IQR: 21.6–66.3 ng/L]), arrhythmias (28.7 ng/L [IQR: 18.7–73.3 ng/L]), and other heart diseases (25.1 ng/L [IQR: 17.9–51.9 ng/L]).

### Diagnostic performance of hs-cTnT concentrations

**Figure 3:**
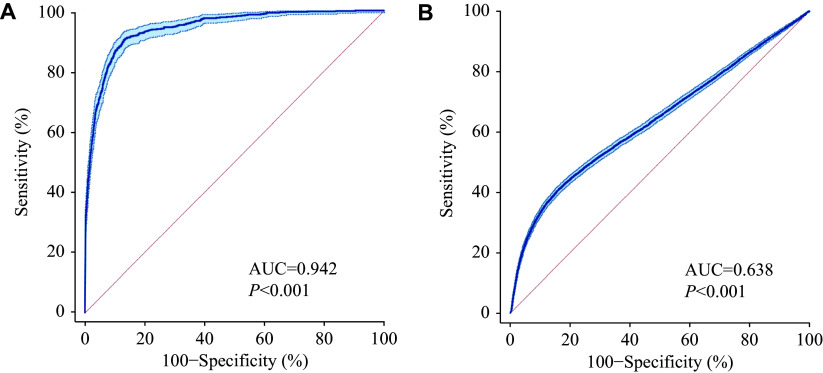
ROC curves demonstrating the diagnostic performance of hs-cTnT with higher cut-off values. As quantified by the area under the ROC curve, the diagnostic accuracy of hs-cTnT is presented above. A: The optimal cut-off value for diagnosing STEMI was 251.9 ng/L, with a sensitivity of 90.7%, specificity of 86.5%, and an AUC of 0.942. B: The optimal cut-off value for diagnosing NSTEMI was 130.5 ng/L, with a sensitivity of 40.9%, specificity of 83.8%, and an AUC of 0.638. Abbreviations: AUC, area under the curve; hs-cTnT, high-sensitivity cardiac troponin T; NSTEMI, non-ST-segment elevation myocardial infarction; ROC, receiver operating characteristic; STEMI, ST-segment elevation myocardial infarction.

**Figure 4:**
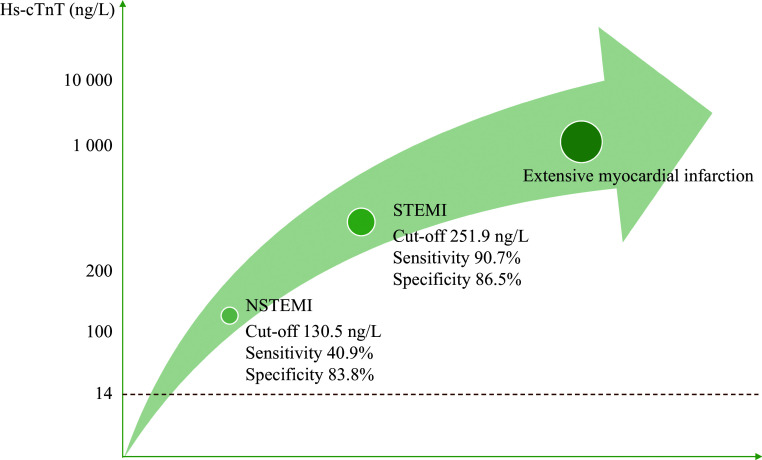
The range for ruling in AMI based on elevated hs-cTnT levels. Abbreviations: AMI, acute myocardial infarction; hs-cTnT, high-sensitivity cardiac troponin T; NSTEMI, non-ST-segment elevation myocardial infarction; STEMI, ST-segment elevation myocardial infarction.

As quantified by the AUC, the diagnostic accuracy of hs-cTnT is shown in ***Fig. 3***. ROC curve analysis revealed that the optimal cut-off value for diagnosing STEMI was 251.9 ng/L, with a sensitivity of 90.7% (95% confidence interval [CI]: 88.5%–92.6%), specificity of 86.5% (95% CI: 86.2%–86.7%), and an AUC of 0.942 (95% CI: 0.940–0.944; *P* < 0.001). The optimal cut-off value for diagnosing NSTEMI was 130.5 ng/L, with a sensitivity of 40.9% (95% CI: 40.1%–41.7%), specificity of 83.8% (95% CI: 83.5%–84.1%), and an AUC of 0.638 (95% CI: 0.635–0.641; *P* < 0.001). The corresponding rule-in range for AMI according to these cut-off values is shown in ***Fig. 4***.

The optimal diagnostic cut-off values for myocardial infarction differed between men and women, with men having a higher hs-cTnT cut-off value than women. As shown in ***Supplementary Fig. 1*** (available online), the optimal cut-off value for diagnosing STEMI in men was 266.0 ng/L, with a sensitivity of 90.1% (95% CI: 87.5%–92.2%), specificity of 86.7% (95% CI: 86.4%–87.0%), and an AUC of 0.941 (95% CI: 0.938–0.943; *P* < 0.001). For women, the cut-off value was 251.9 ng/L, with a sensitivity of 92.1% (95% CI: 86.5%–95.8%), specificity of 87.0% (95% CI: 86.5%–87.4%), and an AUC of 0.948 (95% CI: 0.945–0.951; *P* < 0.001).

As shown in ***Supplementary Fig. 2*** (available online), the optimal cut-off for diagnosing NSTEMI in men was 130.3 ng/L, with a sensitivity of 41.8% (95% CI: 40.8%–42.7%), specificity of 84.1% (95% CI: 83.7%–84.4%), and an AUC of 0.647 (95% CI: 0.643–0.651; *P* < 0.001). For women, the cut-off was 108.2 ng/L, with a sensitivity of 41.4% (95% CI: 39.8%–43.0%), specificity of 80.2% (95% CI: 79.6%–80.8%), and an AUC of 0.615 (95% CI: 0.609–0.622; *P* < 0.001).

***Supplementary Table 1*** (available online) presents the stratified results for NSTEMI patients by sex and age. The age segmentation followed the method by Gore *et al*^[15]^, and the highest cut-offs for diagnosing NSTEMI were observed in older patients (≥ 75 years), with values of 183.1 ng/L for males and 173.5 ng/L for females.

Among the 76411 patients, 12679 (16.6%) had renal dysfunction. Specifically, renal dysfunction was present in 51 (6.3%) of 816 STEMI patients and 1226 (8.3%) of 14686 NSTEMI patients.

In patients with renal dysfunction, the cut-off for diagnosing STEMI was 515.2 ng/L, with sensitivity, specificity, and AUC of 90.2% (95% CI: 78.6%–96.7%), 91.4% (95% CI: 90.9%–91.9%), and 0.941 (95% CI: 0.937–0.945; *P* < 0.001), respectively (***Supplementary Fig. 3A***, available online). In patients with normal renal function, the cut-off for diagnosing STEMI was 247.3 ng/L, with sensitivity, specificity, and AUC of 90.7% (95% CI: 88.4%–92.7%), 86.6% (95% CI: 86.3%–86.8%), and 0.944 (95% CI: 0.942–0.946; *P* < 0.001), respectively (***Supplementary Fig. 3B***, available online).

In patients with renal dysfunction, the cut-off for diagnosing NSTEMI was 182.6 ng/L, with sensitivity, specificity, and AUC of 42.0% (95% CI: 39.2%–44.8%), 83.1% (95% CI: 82.5%–83.8%), and 0.631 (95% CI: 0.623–0.640; *P* < 0.001), respectively (***Supplementary Fig. 4A***, available online). In patients with normal renal function, the cut-off for diagnosing NSTEMI was 90.7 ng/L, with sensitivity, specificity, and AUC of 44.9% (95% CI: 44.1%–45.8%), 81.4% (95% CI: 81.0%–81.7%), and 0.653 (95% CI: 0.649–0.657; *P* < 0.001), respectively (***Supplementary Fig. 4B***, available online).

Additionally, ROC curves were constructed using sex, age, renal dysfunction, and hs-cTnT as combined predictors for diagnosing STEMI and NSTEMI (***Supplementary Fig. 5***, available online). After adjusting for age, sex, and renal dysfunction, the sensitivity, specificity, and AUC were 84.3% (95% CI: 81.6%–86.7%), 89.5% (95% CI: 89.2%–89.7%), and 0.917 (95% CI: 0.915–0.919; *P* < 0.001), respectively, for diagnosing STEMI, and 74.5% (95% CI: 73.8%–75.2%), 45.2% (95% CI: 44.8%–45.6%), and 0.650 (95% CI: 0.647–0.653; *P* < 0.001), respectively, for diagnosing NSTEMI. The adjusted AUC for STEMI was significantly lower than the unadjusted AUC (0.917 *vs.* 0.942; *P* < 0.001), whereas the adjusted AUC for NSTEMI was significantly higher (0.650 *vs.* 0.638; *P* < 0.001) (***Supplementary Fig. 6***, available online).

## Discussion

In the current study, we found that only 34.0% of the elevated hs-cTnT cases were attributed to cardiac diseases, with STEMI accounting for 1.1% and NSTEMI accounting for 19.2%. In this large cohort of Chinese patients with a single elevated hs-cTnT, using higher cut-off values of 251.9 ng/L for STEMI and 130.5 ng/L for NSTEMI demonstrated high accuracy.

Gassenmaier *et al*^[16]^ reported that the specificity of high-sensitivity cardiac troponin I (hs-cTnI) for diagnosing AMI was only approximately 45% when using the cut-off value of the 99th percentile. Similarly, the current study demonstrated that 34.0% of the elevated hs-cTnT cases were caused by cardiac diseases, with AMI accounting for only 20.3%. In addition to AMI, elevated hs-cTnT was observed in other cardiac diseases, such as myocarditis, VHD, CHD, heart failure, cardiomyopathy, and arrhythmias, as well as in non-cardiac diseases, including sepsis, urological disease, rheumatic disease, respiratory disease, endocrine disease, neurological disease, digestive disease, hematological disease, peripheral arterial disease, and cancer. Potential mechanisms of hs-cTnT elevation in these diseases may involve myocardial injury and necrosis, direct myocyte damage, myocardial strain, hypoxemia and hypoperfusion, and inflammatory interstitial remodeling^[17–23]^. Notably, cancer patients accounted for 14.7% of the cohort. Elevated hs-cTnT in cancer patients may be caused by cardiac injury related to cardiotoxicity^[24]^, direct tumor invasion, or infection^[25]^.

The introduction of hs-cTn assays may identify patients with cardiomyocyte injury from causes other than AMI when using the 99th percentile^[5]^. Studies have investigated higher troponin thresholds to rule in AMI^[8,26]^. Mueller-Hennessen *et al*^[26]^ recruited 1282 emergency department (ED) patients with suspected AMI and reported that positive predictive values improved with increasing baseline hs-cTnT cut-offs (from 48.8% for > 14 ng/L to 87.2% for > 60 ng/L) for AMI prediction. The largest study, involving 46092 ED patients with suspected acute coronary syndrome, reported that hs-cTnI rule-in thresholds of 64 ng/L and five-fold URL provided positive predictive values of 59% and 62% with specificities of 96% and 98%, respectively, for diagnosing type 1 myocardial infarction^[8]^. Although these cut-off values had high diagnostic specificity, Thygesen *et al*^[27]^ have emphasized that studies of the diagnostic performance of hs-cTn in more heterogeneous populations are needed because most existing investigations have been conducted in pre-selected ED populations presenting with cardiac symptoms. Correspondingly, the current study enrolled all hospitalized patients with elevated hs-cTnT, including those with acute chest pain and suspected AMI, as well as those who presented with elevated hs-cTnT from any other causes except cardiac surgery. Our results suggest that if the hs-cTnT cut-off is raised to 251.9 ng/L (18-fold of the 99th URL), hs-cTnT alone provides a specificity of 86.5% for diagnosing STEMI, while 130.5 ng/L (10-fold of the 99th URL) yields a specificity of 83.8%for diagnosing NSTEMI, irrespective of clinical symptoms.

It should be noted that STEMI is typically characterized by distinct ECG changes and clinical symptoms. However, in real clinical practice, a subset of STEMI patients presents with atypical symptoms and non-significant ECG changes. Conversely, some patients exhibit clinical symptoms and ST-segment elevations similar to STEMI but are caused by non-myocardial infarction diseases, including myocarditis, pericarditis, and pulmonary embolism. Our optimized hs-cTnT threshold helps provide a more accurate STEMI diagnosis for these patients, particularly in the early stages when further diagnostic tests, such as coronary angiography, are unavailable or not applicable. In addition to patients' symptoms and ECG changes, serial testing with hs-cTnT changes is a reliable strategy for diagnosing AMI and improving diagnostic specificity^[28–29]^.

The sensitivity of the cut-off value for NSTEMI is relatively low, which may be explained by: (1) NSTEMI primarily results from subendocardial myocardial infarction with relatively small infarct areas, leading to mild hs-cTnT elevations and heterogeneous hs-cTnT distribution among patients. This heterogeneity affects the shape of the ROC curve and the determination of the optimal cut-off value. (2) Non-cardiac disease patients with increased hs-cTnT typically show mild elevations that overlap with those of NSTEMI patients, leading to a higher hs-cTnT cut-off for NSTEMI diagnosis, thus reducing its sensitivity. Therefore, for NSTEMI diagnosis, our cut-off value should be adopted to increase diagnostic accuracy and identify high-risk NSTEMI patients (*e.g.*, those with an extensive infarct area). Importantly, it should be noted that a final NSTEMI diagnosis should integrate hs-cTnT levels with the patient's symptoms, including ECG changes and coronary angiography results, *etc*.

Studies have shown that sex differences exist in the AMI diagnosis, regardless of the cut-off values used^[30–33]^. The High-STEAC study included 48282 patients (47% women) and used the hs-cTnI assay with sex-specific thresholds to detect myocardial injury. The study found that this method resulted in a 42% increase in myocardial injury detection in women and a 6% increase in men, identifying five times more female patients with myocardial injury than male patients^[32]^. Gore *et al*^[15]^ found that in three large independent hs-cTnT cohorts, using the Roche hs-cTnT assay, the threshold for men was higher than that for women. Therefore, the implementation of sex-specific thresholds for hs-cTn is of significant importance in diagnosing suspected AMI in both male and female patients. The current study reached a similar conclusion in that, whether diagnosing STEMI or NSTEMI, the cut-off value for men was consistently higher than that for women.

Previous studies have demonstrated that even in the absence of AMI, hs-cTnT levels in patients with renal dysfunction are significantly elevated^[34–35]^. The potential mechanism underlying elevated hs-cTnT in patients with renal dysfunction remains unclear. Possible reasons may include decreased renal clearance and subclinical myocardial injury^[36–37]^. Therefore, using the currently recommended hs-cTnT cut-off (14 ng/L) to diagnose AMI in patients with renal dysfunction reduces diagnostic performance^[38]^. To address this issue, recent studies have shown that increasing the hs-cTnT cut-off values and using renal function-specific hs-cTnT cut-off values may help clinicians diagnose AMI more accurately in patients with renal dysfunction^[34,39]^. Similarly, in the current study, among patients with elevated hs-cTnT, 16.6% had comorbid renal dysfunction. In patients with renal dysfunction, the cut-off values for diagnosing STEMI and NSTEMI are 515.2 ng/L and 182.6 ng/L, respectively, both of which are higher than those in patients with normal renal function.

It should be noted that there is an inverse relationship between specificity and sensitivity. The choice between prioritizing specificity or sensitivity depends on the specific diagnostic requirements as well as the risks associated with false positives and false negatives. Thus, doctors must be aware of this trade-off when evaluating individual patients^[27]^.

Finally, novel biomarkers, including heart fatty acid-binding protein^[40]^ and cardiac myosin-binding protein C^[41]^, are expected to be introduced into clinical practice in the near future to improve the accuracy of diagnosis in combination with troponins.

The strength of this study lies in its being the largest study conducted to date to determine the threshold above the 99th percentile URL of hs-cTnT for diagnosing AMI in heterogeneous populations. Our results may be applied to patients with atypical myocardial ischemia symptoms or non-significant ECG changes, and help provide accurate diagnoses for those requiring prompt diagnosis in emergencies to accelerate the early reperfusion therapies.

As a retrospective study, the diagnosis of all patients was made by the attending physician rather than being adjudicated by an Events Review Committee, and potential bias may arise. However, our study results reflect the real-world diagnosis of patients with elevated hs-cTnT levels, which were based on the guidelines at that time. Additionally, no suitable external database is yet available to verify our conclusions, and our results therefore warrant validation in future prospective studies.

Collectively, in a large cohort of Chinese patients with elevated hs-cTnT levels, the diagnostic performance of hs-cTnT for diagnosing STEMI and NSTEMI was optimized by using higher cut-off values of 251.9 ng/L and 130.5 ng/L, respectively.

## SUPPLEMENTARY DATA

Supplementary data to this article can be found online.
