# Supplementary material for: Using higher cut-off values to diagnose acute myocardial infarction in patients with elevated hs-cTnT
Source: J Biomed Res. 2025 May 21;39(6):564–73. doi: 10.7555/JBR.38.20240364 (PMC12683508; doi:10.7555/JBR.38.20240364)
Supplement: Supplementary file 1 — Supplementary data to this article can be found online. [file jbr-39-6-564-Supplementary.pdf]

Available online at www.jbr-pub.org.cn

Open Access at PubMed Central

JBR

Journal of Biomedical Research, 2025 39(6): 1–3

Supplementary Data

Using higher cut-off values to diagnose acute myocardial infarction in patients with elevated hs-cTnT

Tian Wu[1,△], Jiaqi Chai[1,△], Chunyue Tan[1,△], Zhiwen Tao[1,△], Hui Yong[1], Zhenyu Lin[1], Xiaoxuan Gong[1], Kun Liu[1], Lei Xu[1], Qin Wang[1], Shenqi Jing[2,3,4,5], Jiani Xu[2,5], Hui Zhou[6], Tao Li[6], Liang Yuan[1], Bo Chen[1], Fang Wang[1], Ruxing Wang[7,✉], Yun Liu[3,4,✉], Chunjian Li[1,✉]

[1]Department of Cardiology, the First Affiliated Hospital of Nanjing Medical University, Nanjing, Jiangsu 210029, China;
[2]Center for Data Management, the First Affiliated Hospital of Nanjing Medical University, Nanjing, Jiangsu 210029, China;
[3]Department of Medical Informatics, School of Biomedical Engineering and Informatics, Nanjing Medical University, Nanjing, Jiangsu 211166, China;
[4]Institute of Medical Informatics and Management, Nanjing Medical University, Nanjing, Jiangsu 211166, China;
[5]Jiangsu Province Engineering Research Center of Chronic Disease Big Data Application and Smart Healthcare Service, Nanjing, Jiangsu 210029, China;
[6]Shanghai Synyi Medical Technology Co., Ltd., Shanghai 200000, China;
[7]Department of Cardiology, the Affiliated Wuxi People's Hospital of Nanjing Medical University, Wuxi People's Hospital, Wuxi Medical Center, Nanjing Medical University, Wuxi, Jiangsu 214023, China.

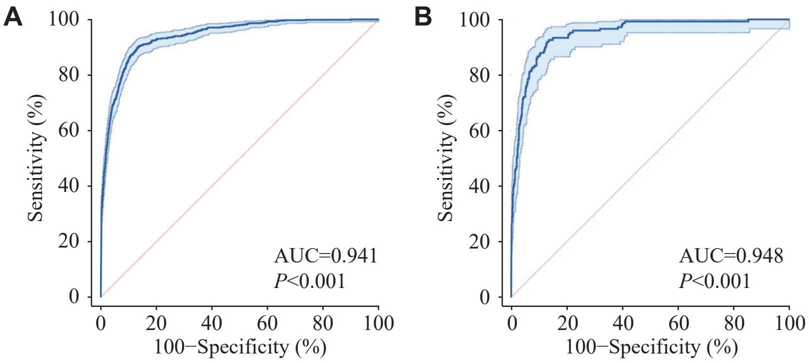

Supplementary Fig. 1 **ROC curves demonstrating the diagnostic performance of hs-cTnT in STEMI.** A: The optimal cut-off level for diagnosing STEMI in males is 266.0 ng/L, with a sensitivity of 90.1% (95% CI: 87.5%–92.2%), specificity of 86.7% (95% CI: 86.4%–87.0%), and an AUC of 0.941 (95% CI: 0.938–0.943, P < 0.001). B: The optimal cut-off level for diagnosing STEMI in females is 251.9 ng/L, with a sensitivity of 92.1% (95% CI: 86.5%–95.8%), specificity of 87.0% (95% CI: 86.5%–87.4%), and an AUC of 0.948 (95% CI: 0.945–0.951, P < 0.001). Abbreviations: AUC, area under the curve; CI, confidence interval; hs-cTnT, high-sensitivity cardiac troponin T; ROC, receiver operating characteristic; STEMI, ST-segment elevation myocardial infarction.

△These authors contributed equally to this work.

✉Corresponding authors: Ruxing Wang, Department of Cardiology, the Affiliated Wuxi People's Hospital of Nanjing Medical University, Wuxi People's Hospital, Wuxi Medical Center, Nanjing Medical University, 299 Qingyang Road, Wuxi, Jiangsu 214023, China. E-mail: ruxingw@njmu.edu.cn; Yun Liu, Department of Medical Informatics, School of Biomedical Engineering and Informatics, Nanjing Medical University, 101 Longmian Avenue, Nanjing, Jiangsu 211166, China. E-mail: liuyun@njmu.edu.cn; Chunjian Li, Department of Cardiology, the First Affiliated Hospital of Nanjing Medical University, 300 Guangzhou Road, Nanjing, Jiangsu 210029, China. E-mail: lijay@njmu.edu.cn.

Received: 28 October 2024; Revised: 08 May 2025; Accepted: 18 May 2025; Published online: 21 May 2025

CLC number: R542.2, Document code: A

The authors reported no conflict of interests.

This is an open access article under the Creative Commons Attribution (CC BY 4.0) license, which permits others to distribute, remix, adapt and build upon this work, for commercial use, provided the original work is properly cited.

© 2025 by Journal of Biomedical Research.

https://doi.org/10.7555/JBR.38.20240364

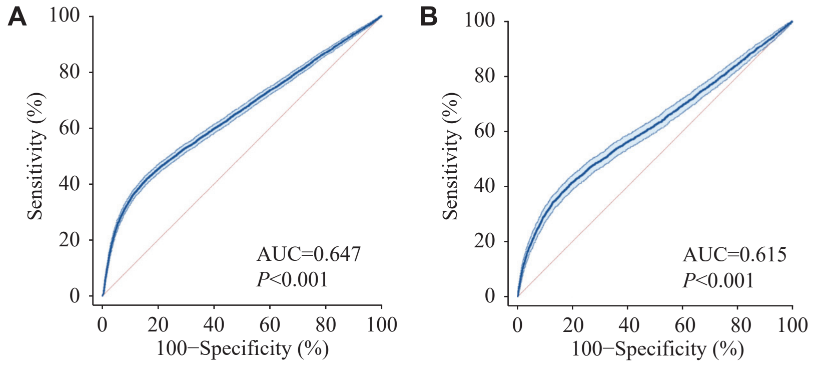

Supplementary Fig. 2 **ROC curves demonstrating the diagnostic performance of hs-cTnT in NSTEMI.** A: The optimal cut-off level for diagnosing NSTEMI in males is 130.3 ng/L, with a sensitivity of 41.8% (95% CI: 40.8%–42.7%), specificity of 84.1% (95% CI: 83.7%–84.4%), and an AUC of 0.647 (95% CI: 0.643–0.651, $P < 0.001$). B: The optimal cut-off level for diagnosing NSTEMI in females is 108.2 ng/L, with a sensitivity of 41.4% (95% CI: 39.8%–43.0%), specificity of 80.2% (95% CI: 79.6%–80.8%), and an AUC of 0.615 (95% CI: 0.609–0.622, $P < 0.001$). Abbreviations: AUC, area under the curve; CI, confidence interval; hs-cTnT, high-sensitivity cardiac troponin T; NSTEMI, non-ST-segment elevation myocardial infarction; ROC, receiver operating characteristic.

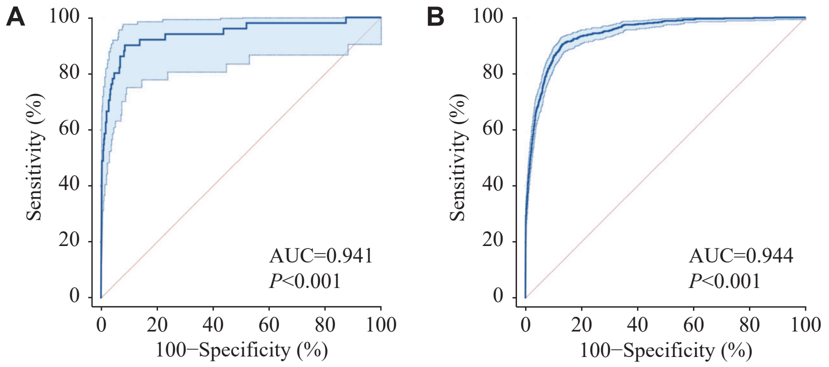

Supplementary Fig. 3 **ROC curves demonstrating the diagnostic performance of hs-cTnT in STEMI.** A: The optimal cut-off level for diagnosing STEMI in patients with renal dysfunction is 515.2 ng/L, with a sensitivity of 90.2% (95% CI: 78.6%–96.7%), specificity of 91.4% (95% CI: 90.9%–91.9%), and an AUC of 0.941 (95% CI: 0.937–0.945, $P < 0.001$). B: The optimal cut-off level for diagnosing STEMI in patients with normal renal function is 247.3 ng/L, with a sensitivity of 90.7% (95% CI: 88.4%–92.7%), specificity of 86.6% (95% CI: 86.3%–86.8%), and an AUC of and 0.944 (95% CI: 0.942–0.946, $P < 0.001$). Abbreviations: AUC, area under the curve; CI, confidence interval; hs-cTnT, high-sensitivity cardiac troponin T; ROC, receiver operating characteristic; STEMI, ST-segment elevation myocardial infarction.

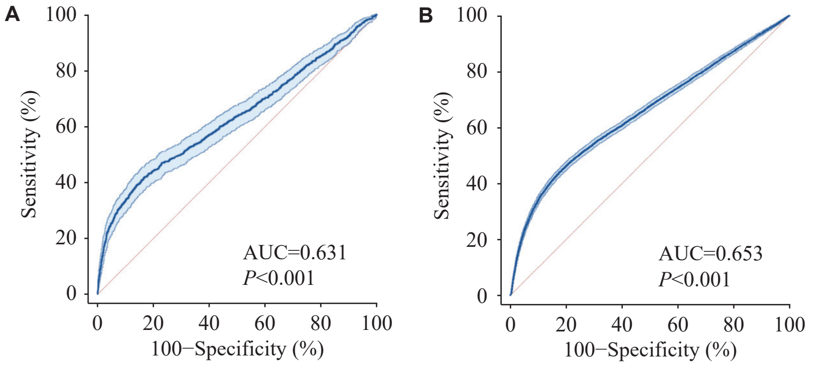

Supplementary Fig. 4 **ROC curves demonstrating the diagnostic performance of hs-cTnT in NSTEMI.** A: The optimal cut-off level for diagnosing NSTEMI in patients with renal dysfunction is 182.6 ng/L, with a sensitivity of 42.0% (95% CI: 39.2%–44.8%), specificity of 83.1% (95% CI: 82.5%–83.8%), and an AUC of and 0.631 (95% CI: 0.623–0.640, $P < 0.001$). B: The optimal cut-off level for diagnosing NSTEMI in patients with normal renal function is 90.7 ng/L, with a sensitivity of 44.9% (95% CI: 44.1%–45.8%), specificity of 81.4% (95% CI: 81.0%–81.7%), and an AUC of and 0.653 (95% CI: 0.649–0.657, $P < 0.001$). Abbreviations: AUC, area under the curve; CI, confidence interval; hs-cTnT, high-sensitivity cardiac troponin T; NSTEMI, non-ST-segment elevation myocardial infarction; ROC, receiver operating characteristic.

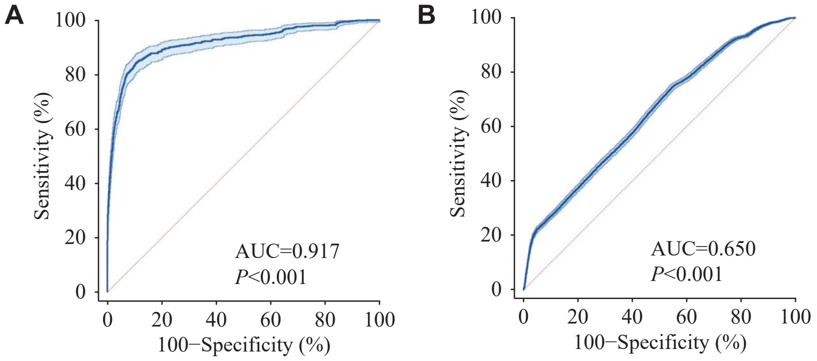

Supplementary Fig. 5 **ROC curves demonstrating the diagnostic performance of adjusted hs-cTnT in AMI.** A: For diagnosing STEMI, the sensitivity, specificity, and AUC changed to 84.3% (95% CI: 81.6%–86.7%), 89.5% (95% CI: 89.2%–89.7%), and 0.917 (95% CI: 0.915–0.919, P < 0.001). B: For diagnosing NSTEMI, the sensitivity, specificity, and AUC changed to 74.5% (95% CI: 73.8%–75.2%), 45.2% (95% CI: 44.8%–45.6%), and 0.650 (95% CI: 0.647–0.653, P < 0.001). Abbreviations: AMI, acute myocardial infarction; AUC, area under the curve; CI, confidence interval; hs-cTnT, high-sensitivity cardiac troponin T; NSTEMI, non-ST-segment elevation myocardial infarction; ROC, receiver operating characteristic; STEMI, ST-segment elevation myocardial infarction.

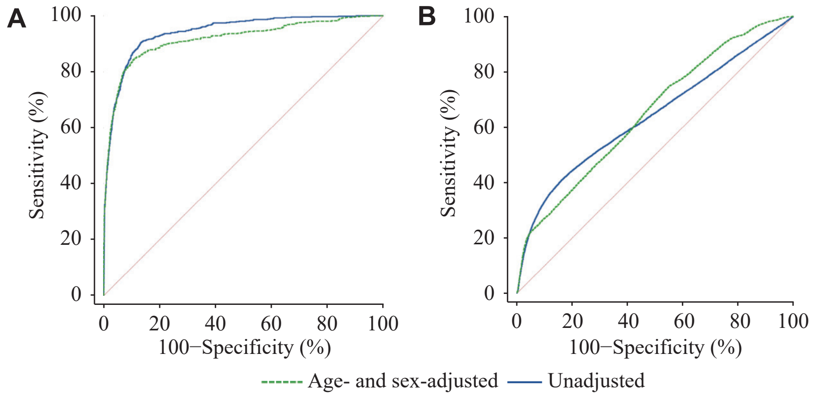

Supplementary Fig. 6 **Comparisons between ROC curves demonstrating the diagnostic performance of hs-cTnT in AMI.** A: Comparisons between ROC curves in STEMI. B: Comparisons between ROC curves in NSTEMI. Abbreviations: AMI, acute myocardial infarction; hs-cTnT, high-sensitivity cardiac troponin T; NSTEMI, non-ST-segment elevation myocardial infarction; ROC, receiver operating characteristic; STEMI, ST-segment elevation myocardial infarction.

Supplementary Table 1 **Stratified analysis results of NSTEMI patients by age and sex**

Sex and age	Number	Age (years, mean±SD)	Hs-cTnT (ng/L, median [IQR])	Cut-off (ng/L, median [IQR])	Sensitivity (%, median [IQR])	Specificity (%, median [IQR])	AUC
Male (< 50 years)	1 063	43.2±5.2	211.7 (36.2–1 360.0)	127.1	57.9 (54.8–60.8)	75.1 (74.0–76.3)	0.675
Male (≥ 50, < 65 years)	3 426	57.8±4.3	111.8 (26.7–872.1)	129.6	48.3 (46.6–50.0)	81.9 (81.1–82.6)	0.676
Male (≥ 65, < 75 years)	3 320	69.4±2.9	56.0 (21.1–471.9)	78.9	45.1 (43.4–46.8)	81.7 (80.9–82.3)	0.664
Male (≥ 75 years)	3 245	81.6±5.3	44.2 (21.5–291.9)	183.1	29.6 (28.0–31.2)	90.0 (89.5–90.5)	0.602
Female (< 50 years)	109	44.1±5.4	110.2 (30.5–620.1)	101.1	53.2 (43.4–62.8)	70.3 (68.6–72.0)	0.636
Female (≥ 50, < 65 years)	719	58.9±4.0	96.2 (30.2–549.4)	112.3	47.7 (44.0–51.4)	78.2 (76.9–79.4)	0.654
Female (≥ 65, < 75 years)	1 254	69.7±2.8	63.4 (21.8–438.5)	107.8	42.3 (39.5–45.1)	82.1 (81.1–83.2)	0.631
Female (≥ 75 years)	1 550	81.8±5.6	46.7 (21.3–314.5)	173.5	32.0 (29.7–34.4)	89.8 (89.1–90.5)	0.607
Total	14 686	68.1±12.6	67.2 (22.9–548.9)	130.5	40.9 (40.1–41.7)	83.8 (83.5–84.1)	0.638

Abbreviations: AUC, area under the curve; hs-cTnT, high-sensitivity cardiac troponin T; IQR, interquartile range; NSTEMI, non-ST-segment elevation myocardial infarction; SD, standard deviation.
